# HAAN: Learning a Hierarchical Adaptive Alignment Network for Image-Text Retrieval

**DOI:** 10.3390/s23052559

**Published:** 2023-02-25

**Authors:** Shuhuai Wang, Zheng Liu, Xinlei Pei, Junhao Xu

**Affiliations:** 1 School of Computer Science and Technology, Shandong University of Finance and Economics, Jinan 250014, China; 2 Shandong Provincial Key Laboratory of Digital Media Technology, Jinan 250014, China

**Keywords:** image-text retrieval, global-level alignment, local-level alignment, adaptive weighted loss

## Abstract

Image-text retrieval aims to search related results of one modality by querying another modality. As a fundamental and key problem in cross-modal retrieval, image-text retrieval is still a challenging problem owing to the complementary and imbalanced relationship between different modalities (i.e., Image and Text) and different granularities (i.e., Global-level and Local-level). However, existing works have not fully considered how to effectively mine and fuse the complementarities between images and texts at different granularities. Therefore, in this paper, we propose a hierarchical adaptive alignment network, whose contributions are as follows: (1) We propose a multi-level alignment network, which simultaneously mines global-level and local-level data, thereby enhancing the semantic association between images and texts. (2) We propose an adaptive weighted loss to flexibly optimize the image-text similarity with two stages in a unified framework. (3) We conduct extensive experiments on three public benchmark datasets (Corel 5K, Pascal Sentence, and Wiki) and compare them with eleven state-of-the-art methods. The experimental results thoroughly verify the effectiveness of our proposed method.

## 1. Introduction

In the information age, images and texts are the two most significant data for understanding the natural world. Therefore, designing efficient retrieval methods has become an essential prerequisite for obtaining multi-modal information. For example, when users are interested in an image, they can use it to retrieve related texts through effective image-text retrieval technologies, and vice versa. However, the heterogeneous properties of images and texts make the mutual retrieval between them quite challenging. Therefore, to realize high-precision image-text retrieval [1], the heterogeneous gap [2] should be well solved.

To bridge this gap, mainstream researches on image-text retrieval tend to put stress on learning about the two different patterns of common embedded in space, which can be roughly divided into (1) global-level alignment methods [3,4,5,6] and (2) local-level alignment methods [7,8,9,10,11]. To be specific, the goal of global-level alignment methods is to map the whole image and text into a common potential embedded space and further calculate the image-text similarity. However, there actually exist visual objects and textual key words in images and text. It is of great importance to take advantage of their local-level features when calculating the image-text similarity. To find solutions for these problems, a number of local-level alignment methods that can learn the relationship between features of image patches and words have been proposed. For example, SCAN [7] adopts a stacked cross-attention module to conduct local-level alignment between visual objects and textual key words to capture more comprehensive cross-modal associations. Besides, most image-text retrieval methods usually use triplet loss [12] to optimize the parameters of models and achieve better image-text retrieval performance.

Although all these methods turn out to perform well, we believe that there are still two limitations that prevent them from achieving better retrieval performance. That is, the motivations of this work lie in the following two aspects:

- Global-level features contain general information about images or texts, while local-level features focus on their details. However, most existing methods only take a single-level feature into account to calculate the image-text similarity, while ignoring the different roles and effects of different features. Therefore, we propose to fully explore the integration of hierarchical alignment features so that image-text retrieval can provide a more accurate retrieval result.
- Using triplet loss for optimization will bring about 2 disadvantages. Firstly, the training samples are constructed into triples, and then produce a large number of redundant pairs containing a small amount of information. Randomly sampling these training pairs will result in slow convergence. Secondly, the triplet loss optimizes all training pairs with the same strength, which fails to fully use the training samples with differentiation and will lead to performance degradation. So we suggest considering two iterative stages of sampling and weighting in the design of the loss function. To filter out redundant information, we only select some representative samples: (1) to generate positive pairs, samples that are farther away from the anchor are chosen, and (2) to generate negative pairs, samples that are closer to the anchor are utilized. For better performance, we assign different weights to different positive pairs and negative pairs by fully exploiting the discriminative training samples.

Inspired by the above discussions, our research suggests a Hierarchical Adaptive Alignment Network (HAAN) for Image-text Retrieval, which combines the hierarchical alignment and the adaptive optimization together to enhance the performance of image-text retrieval.

**Figure 1:**
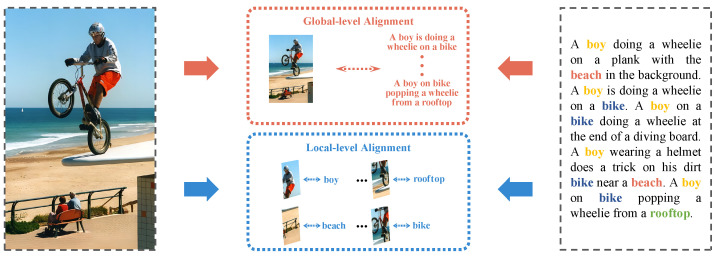
An example of hierarchical alignment for image-text correlation learning, which not only explores global-level alignment between the whole image and text, but also considers the local-level alignment between image blocks and keywords.

To be specific, as shown in Figure 1, when it comes to matching an image with a text, we integrate global-level alignment and local-level alignment together to learn better image-text correlation. For global-level alignment, it refers to learning the similarity of the whole image and text based on global-level features. Different from global-level alignment, local-level alignment refers to learning the similarity of image blocks and keywords based on local-level features. Then, we carry out an adaptive optimization for global-level similarity and local-level similarity and fuse them together. The main contributions we made in this paper can be concluded in the following points:

- A hierarchical adaptive alignment network is proposed to innovatively exploit multi-level clues within images and texts, which fully explores the integration of the global-level and local-level features to improve the performance of image-text retrieval.
- We put forward an adaptive weighted loss method to accurately optimize image-text similarity through two stages. In stage 1, we select positive and negative pairs that contain rich information to accelerate convergence. In stage 2, we design different weights for different pairs to achieve better performance.
- Based on extensive experiments on three widely-used benchmark datasets, it is shown that compared with several state-of-the-art image-text retrieval approaches, the method we proposed tends to achieve the best performance.

The organizational structure of the following content is as follows: Firstly, we review the generation related to this study in Section 2 briefly. Then, Section 3 details the interpretation of our method. Afterward, we conduct a series of related experiments to verify and analyze the proposed method in Section 4. Lastly, Section 5 provides a summary of the whole paper.

## 2. Related Work

In this section, we briefly review representative methods for image-text retrieval. Specifically, we discuss the mainstream methods in Section 2.1, and then discuss the application of metric learning for image-text retrieval in Section 2.2.

### 2.1. Image-Text Retrieval

In order to bridge the heterogeneous gap between images and texts, the mainstream of existing methods focuses on building a common embedding space to calculate the similarity between different modalities. Learning image-text correlation with global-level or local-level features is very commonly seen in previous works.

#### 2.1.1. Image-Text Retrieval Using Global-Level Features

Various image-text retrieval methods mainly concentrate on global-level information to achieve image and text matching, which is embodied in capturing the global-level visual-textual correspondence. In 2017, breakthroughs were made in research by Faghri et al. [5], where the scholars encoded the image by a CNN, and a GRU-based text encoder to extract the feature of sentences is proposed. Wang et al. [4] put forward a method with a two-branch network to analyze the correspondence between different modalities in a creative way. By incorporating the generative model into image-text embedding, Gu et al. [3] conducted research to explore richer representations. According to the research conducted by Wen et al. [6], scholars put forward a cross-memory network with pair discrimination, by which the common knowledge between image and text modalities is captured. Although these methods have made great achievements, the local-level alignment between different modalities is ignored.

#### 2.1.2. Image-Text Retrieval Using Local-Level Features

The image-text retrieval methods depending on local-level features also become predominant in recent years. It aligns differentiated image areas with corresponding index terms that describe certain objects. To find out the latent region-word correspondences, Lee et al. [7] proposed a stacked cross-attention module. A bi-directional focal attention network was presented by Liu et al. [8]. In this network, image-text alignment can be analyzed through an emphasis on relevant fragments. Aiming at the relationship-enhanced visual features, a visual reasoning network introduced by Li et al. [9] performed successfully as well. Additionally, Chen et al. [11] put forward an iterative matching scheme, which worked creatively for the recurrent attention memory module designed to capture the image-text correspondences it owned. Zhang et al. [10] proposed a novel negative-aware attention framework, in which both the positive influence of matched fragments and the negative consequence from mismatched fragments were taken into account. Scholars made full use of these together to deduce image-text similarity.

In conclusion, most current methods only take single-level information into consideration when calculating the image-text similarity. It is worth noting that, different from previous studies mentioned above, we entirely search and fuse the global- and local-level information in image and text, yielding more semantic information for the sake of image-text retrieval.

### 2.2. Metric Learning of Image-Text Retrieval

Recently, metric learning has become a hot topic, which is designed to use a loss function to measure similarity and then improve the method’s performance by pulling semantically relevant samples closer and pushing apart semantically irrelevant samples. A triplet loss was proposed by Schroff et al. [12] which attempts to explore a feature space, where positive samples stayed closer and negative samples stayed farther to anchors. When it comes to the image-text retrieval task with n image-text pairs, the time complexity of triplet loss was $O(N^{3})$, and it was not feasible to traverse all sample pairs during training. To conclude, selecting typical samples attaches great significance to metric learning.

A deep coupled metric learning proposed by Liong et al. [13] can manage to reduce the modality map by two nonlinear transformations. In research by Faghri et al. [5], a variant triplet loss for image-text matching was introduced while improved results were also reported. Xu et al. [14] proposed a modality classifier in their studies, which is utilized to make sure the transformed features showing statistically indistinguishable. Nevertheless, the methods we have discussed all share a balanced view of positive and negative pairs.

In conclusion, previous works as mentioned above can not precisely distinguish samples based on levels of significance. Some works even treat the optimization of various samples equally, resulting in poor retrieval performance and slow convergence. In this paper, we suggest an adaptive weighted loss that integrates pairs mining and pairs weighting together in a unified framework to optimize image-text similarity more accurately.

## 3. Our Method

**Figure 2:**
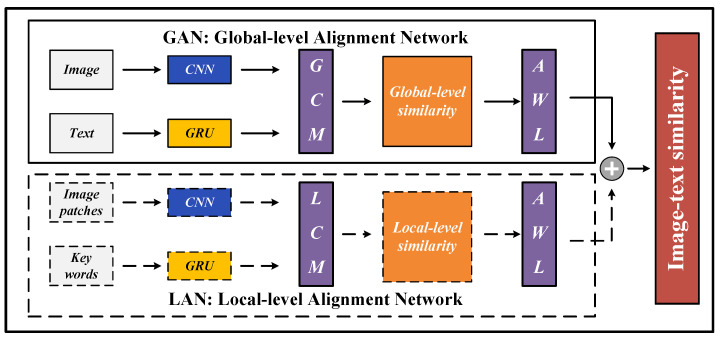
The pipeline diagram of the whole solution of HAAN.

This section aims to offer an interpretation of the method we proposed. Firstly, the general framework and feature extraction are explained in Section 3.1 and Section 3.2, respectively. Next, the Global-level Image-Text Similarity Computation Module (GCM) and the Local-level Image-Text Similarity Computation Module (LCM) are elaborated in Section 3.3. Finally, the Adaptive Weighted Loss (AWL) is explained detailedly in Section 3.4. In addition, all the important notations are listed in Table 1. In Figure 2, we provide the pipeline diagram of the whole solution, which illustrates the calculation process of HAAN. In general, solid lines represent the global-level data streams, while dotted lines represent the local-level data streams. These two types of data streams are optimized by AWL and then fused together with linear weights to obtain the image-text similarity. For the global-level data streams, we extract the features of the whole images and the whole texts using CNN and Bi-GRU, respectively, and then calculate the cosine similarity. For local-level data streams, we extract the features of image patches and words with CNN and Bi-GRU as well, and estimate the similarity between them using the cross-attention mechanism.

### 3.1. Framework of HAAN

The HAAN consists of a Global-level Alignment Network (GAN) and a Local-level Alignment Network (LAN) based on GCM and LCM, respectively. Note that, the proposed AWL τ(·) is used to optimize the image-text similarity matrix in each subnetwork.

Firstly, we define the global-level objective function ${ℶ}^{C}$ in GAN as follows:

(1) $${ℶ}^{C}=\tau \left(L^{C},\vartheta^{C}\right)$$

where the global-level similarity matrix $L^{C}$ is calculated by GCM and $\vartheta^{C}$ are the parameters of GAN. Then, we derive the gradient of the function ${ℶ}^{C}$ with respect to $\vartheta^{C}$ as follows:

(2) $$\frac{\partial \tau (L^{C},\vartheta^{C})}{\partial \vartheta^{C}}=\sum_{i=1}^{B}\sum_{j=1}^{B}\frac{\partial \tau (L^{C},\vartheta^{C})}{\partial L_{ij}^{C}}\frac{\partial L_{ij}^{C}}{\partial \vartheta^{C}}$$

where *B* is the batch size. Afterwards, the optimal global-level similarity matrix ${\tilde{L}}^{C}$ is solved as follows:

(3) $${\tilde{L}}^{C}=\underset{L^{C}}{\mathrm{arg min}}\tau \left(L^{C},\vartheta^{C}\right)$$

Secondly, the local-level objective function ${ℶ}^{F}$ in LAN is represented as follows:

(4) $${ℶ}^{F}=\tau \left(L^{F},\vartheta^{F}\right)$$

where the local-level similarity matrix $L^{F}$ is calculated by LCM and $\vartheta^{F}$ are the parameters of LAN. Similarly, we get the optimal local-level similarity matrix ${\tilde{L}}^{F}$ by deriving the gradient of the function ${ℶ}^{F}$ with respect to $\vartheta^{F}$ as follows.

(5) $$\frac{\partial \tau (L^{F},\vartheta^{F})}{\partial \vartheta^{F}}=\sum_{i=1}^{B}\sum_{j=1}^{B}\frac{\partial \tau (L^{F},\vartheta^{F})}{\partial L_{ij}^{F}}\frac{\partial L_{ij}^{F}}{\partial \vartheta^{F}}$$

Thus, the optimal global-level similarity matrix ${\tilde{L}}^{F}$ is obtained as follows.

(6) $${\tilde{L}}^{F}=\underset{L^{F}}{\mathrm{arg min}}\tau \left(L^{F},\vartheta^{F}\right)$$

Finally, in Equation (7) global-level and local-level optimal similarity matrices are fused by a linear weighted fusion strategy.

(7) $$\tilde{L}=\upsilon_{1}{\tilde{L}}^{C}+\upsilon_{2}{\tilde{L}}^{F}$$

where $\tilde{L}$ is used to perform image-text retrieval, and $\upsilon_{1},\upsilon_{2}$ represent the fusion coefficients.

**Figure 3:**
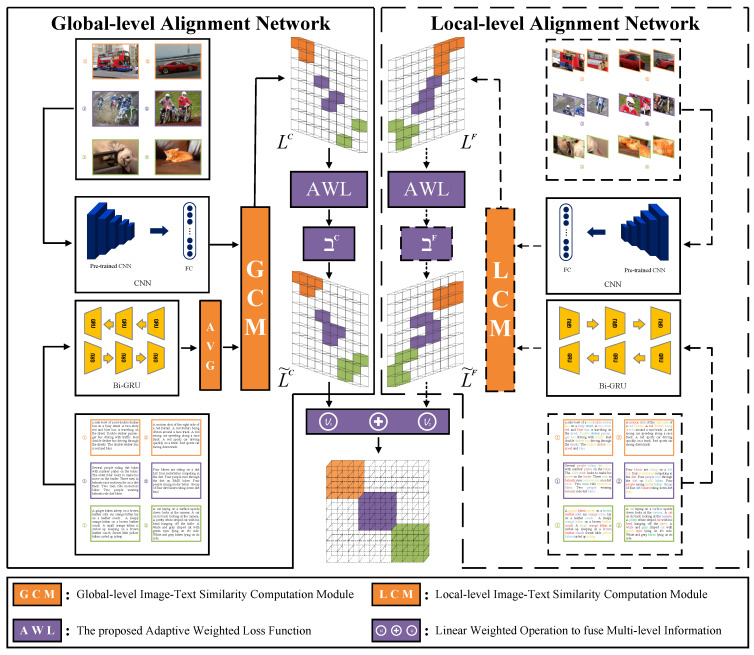
The overall framework of HAAN method. In general, it is composed of a global-level alignment network (shown at the left half) and a local-level alignment network (shown at the right half). Among them, GCM and LCM are designed to work out the global- and local-level image-text similarity matrix. Besides, AWL is responsible for adaptively optimizing the similarity matrix before linear weighted fusion.

The framework of HAAN is shown in Figure 3. Noticeably, the features of the images and texts are extracted by CNN and Bi-GRU respectively, and then sent to GCM and LCM to obtain the corresponding similarity matrices. Through the optimization of AWL, the optimal similarity matrices are further obtained and fused. In addition, solid lines and dotted lines are used to represent the global-level data streams and the local-level data streams, respectively.

**Figure 4:**
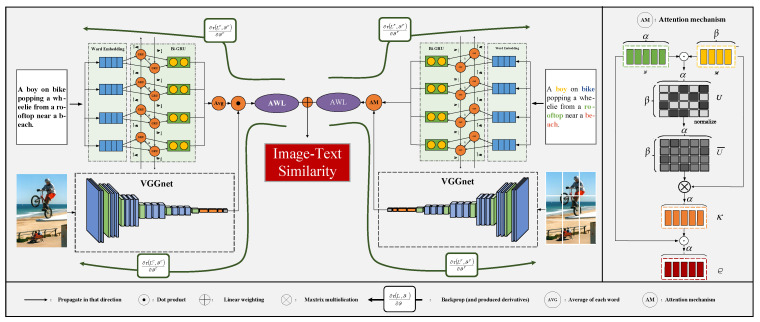
The deep learning network architectures of HAAN.

To further illustrate details of HAAN, the deep learning network architectures of it are described in Figure 4.

Particularly, the left part and the right part of Figure 4 are corresponding to the GAN and LAN, respectively. In the left part, we input the one-hot vector of each word to Bi-GRU, and the feature of the whole text is solved by computing the average of word features that are output from Bi-GRU, meanwhile, we utilize VGGnet to extract image features. Afterward, the initial image-text similarity is calculated by doing a dot product between the feature vectors of images and texts, and then it is optimized by AWL. Furthermore, the right part is just similar to the left one, except that the fine-grained keywords and image patches are input to LAN. Afterward, the image-to-text attention mechanism is used to compute the initial image-text similarity. In the end, the optimized global-level similarity and local-level similarity are linearly weighted and fused to obtain the final image-text similarity. Additionally, the gradient descent method used in HAAN is clearly presented as well.

### 3.2. Feature Extraction

Given a dataset $\left\{{\left(I_{p},T_{q}\right)}_{p,q=1}^{N}\right\}$ consisting of *N* pairs of images and texts. Besides, there are α patches in image $I_{p}$ and β words in text $T_{q}$. We first extract global-level and local-level features and then encode them into a common embedding space. Additionally, we use Convolutional Neural Network (CNN) for image feature extraction and Bidirectional GRU (Bi-GRU) for text feature extraction.

#### 3.2.1. Global-Level Feature Extraction

In our work, the global-level feature vectors of $I_{p}$ and $T_{q}$ are donated as $n_{p}^{C}\in R^{1024}$ and $m_{q}^{C}\in R^{1024}$, respectively. The detailed process of global-level feature extraction is explained as follows.

Global-level feature of image. We extract the feature vector $u_{p}^{C}\in R^{4096}$ of image $I_{p}$ from FC-7 of pre-trained VGGnet [15]. Then, the feature vector is projected to the 1024-d embedding space through the fully connected layer as Equation (8).

(8) $$n_{p}^{C}=K^{C}*u_{p}^{C}+b^{C}$$

where $K^{C}$ and $b^{C}$ refer to the weight matrix and bias term to be optimized, and $n_{p}^{C}\in R^{1024}$ is the image global-level feature vector.

Global-level feature of text. Firstly, the words in each text are represented as one-hot vectors, and then they are embedded into 300-d feature space. Formally, the *y*th word in $T_{q}$ is donated as $k_{q,y}\in R^{300}$. Secondly, original textual features are mapped into a 1024-d embedding space for making a direct comparison between images and texts. As shown in Equation (9), we use the Bi-GRU to model the textual context of text $T_{q}$ from both two different directions.

(9) $$\begin{array}{c} {\vec{h}}_{q,y}=\vec{GRU}\left(k_{q,y},{\vec{h}}_{q,y-1}\right)\\ {\overset{\leftarrow }{h}}_{q,y}=\overset{\leftarrow }{GRU}\left(k_{q,y},{\overset{\leftarrow }{h}}_{q,y+1}\right) \end{array}$$

where ${\vec{h}}_{q,y}$, ${\overset{\leftarrow }{h}}_{q,y}$ indicate the forward and backward hidden states of Bi-GRU. The feature vector $m_{q,y}$ for *y*th word in text $T_{q}$ is computed with $m_{q,y}=\left({\vec{h}}_{q,y}+{\overset{\leftarrow }{h}}_{q,y}\right)/2$. Finally, the global-level feature vector of $T_{q}$ is obtained by calculating the average value of all word vectors: $m_{q}^{C}=\frac{1}{\beta }\sum_{y=1}^{\beta }m_{q,y}$, $m_{q}^{C}\in R^{1024}$.

#### 3.2.2. Local-Level Feature Extraction

Additionally, the local-level feature vectors of $I_{p}$ and $T_{q}$ are donated as $N_{p}^{F}=\{n_{p,x}^{F}|x=$ $1,\ldots ,\alpha ,n_{p,x}^{F}\in R^{1024}\}$ and $M_{q}^{F}=\left\{m_{q,y}^{F}\right|y=1,\ldots ,\beta ,m_{q,y}^{F}\in R^{1024}\}$, respectively. The detailed process of local-level feature extraction is as follows.

Local-level feature of image. Similarly, we also extract the local-level feature vectors of $I_{p}$ by VGGnet, which are donated as $U_{p}^{F}=\left\{u_{p,x}^{F}|x=1,\cdots ,\alpha ,u_{p,x}^{F}\in R^{4096}\right\}$. Then, they are also projected into the 1024-d embedding space through the fully connected layer like Equation (8), and $N_{p}^{F}=\left\{n_{p,x}^{F}|x=1,\cdots ,\alpha ,n_{p,x}^{F}\in R^{1024}\right\}$ donate the local-level feature vectors of $I_{p}$.

Local-level feature of text. Like Equation (9), we utilize the Bi-GRU to extract the feature of each word in each text: $m_{q,y}=\left({\vec{h}}_{q,y}+{\overset{\leftarrow }{h}}_{q,y}\right)/2$. Thus, the local-level feature vectors of $T_{q}$ are represented as $M_{q}^{F}=\left\{m_{q,y}^{F}\right|y=1,\ldots ,\beta ,m_{q,y}^{F}\in R^{1024}\}$.

### 3.3. Image-Text Similarity Calculation

GCM and LCM are used to calculate the image-text similarity at the global level and the local level, respectively. Notably, to learn local-level correlations between images and texts more accurately, the attention mechanism is employed in LCM, which can fully aggregate local-level matches between patches and words.

#### 3.3.1. GCM: Global-Level Image-Text Similarity Computation Module

The global-level feature vectors $n_{p}^{C}$ and $m_{q}^{C}$ are input to GCM to calculate the global-level image-text similarity as follows.

(10) $$L^{C}\left(p,q\right)=\frac{{n_{p}^{C}}^{T}m_{q}^{C}}{\|n_{P}^{C}\left\|\cdot \right\|m_{q}^{C}\|}$$

#### 3.3.2. LCM: Local-Level Image-Text Similarity Computation Module

Correspondingly, the local-level feature vectors $N_{p}^{F}$ and $M_{q}^{F}$ are fed into LCM to obtain the local-level image-text similarity. In LCM, we learn a cross-attention embedding space to figure out the latent alignment relationship between local-level features of images and texts.

Firstly, we calculate the cosine similarity matrix *U* with $N_{p}^{F}$ and $M_{q}^{F}$ to reveal the associations between all possible patch-word pairs. Equation (11) represents the association between the *x*th patch and the *y*th word. Then we normalize *U* according to its column dimension as Equation (12).

(11) $$U_{xy}=\frac{n_{p,x}^{T}m_{q,y}}{\|n_{p,x}\left\|\cdot \right\|m_{q,y}\|},\forall x\in \left[1,\alpha \right],\forall y\in \left[1,\beta \right]$$

(12) $${\bar{U}}_{xy}=\frac{relu(U_{xy})}{\sqrt{\sum_{x=1}^{\alpha }relu{\left(U_{xy}\right)}^{2}}},relu\left(n\right)=\max\left(0,n\right)$$

Afterward, for the *x*th patch in $I_{p}$, the text-context feature vector $\kappa_{p,x}$ is defined as a weighted integration with representations of words through the attention mechanism. Furthermore $\kappa_{p,x}$ is computed using Equation (13).

(13) $$\kappa_{p,x}=\sum_{y=1}^{\beta }\left(\frac{exp(\lambda {\bar{U}}_{xy})}{\sum_{y=1}^{\beta }exp\left(\lambda {\bar{U}}_{xy}\right)}\right)*m_{q,y}$$

where λ is performed as the temperature-inverse parameter for the softmax function, and adjusts the smoothness of the attention distribution. In order to evaluate the importance of each image patch in a given text context, we compute a cosine function as Equation (14).

(14) $$\varrho \left(n_{p,x},\kappa_{p,x}\right)=\frac{n_{p,x}^{T}\kappa_{p,x}}{\|n_{p,x}\left\|\cdot \right\|\kappa_{p,x}\|}$$

The similarity $L^{F}\left(p,q\right)$ is obtained as Equation (15) by averaging all relevance scores.

(15) $$L^{F}\left(p,q\right)=\frac{1}{\alpha }\sum_{x=1}^{\alpha }\varrho \left(n_{p,x},\kappa_{p,x}\right)$$

Lastly, the global-level image-text similarity matrix $L^{C}$, as well as the local-level image-text similarity matrix $L^{F}$, are figured out and then optimized by the proposed AWL.

### 3.4. The Adaptive Weighted Loss

The proposed AWL is used to optimize the image-text similarity matrix more precisely in two stages, which not only has the characteristic of fast convergence but also adaptively optimizes the image-text similarity to improve the performance of image-text retrieval.

#### 3.4.1. Image-Text Pairs Sampling

Given an image or a text as an anchor, the texts or images from the same class are used to form positive pairs with it, while the texts or images from the different classes are exploited to construct negative pairs. Notably, the least similar positive pair and the most similar negative pair are used to perform informative pairs sampling.

Formally, assume that $s_{i}$ refers to an anchor and $s_{j}$ is a candidate, and $s_{i}$, $s_{j}$ belong to class $h_{i}$ and $h_{j}$, respectively. If $s_{i}$, $s_{j}$ are from the same class, i.e., $h_{i}=h_{j}$, they are a positive pair and the similarity between them is donated as $L_{ij}^{+}$. Besides, the similarity between a negative pair is donated as $L_{ij}^{-}$ when $h_{i}\neq h_{j}$. In our work, we propose to sample informative positive and negative pairs through the following conditions, respectively.

(16) $$L_{ij}^{+}<\max_{}L_{ik}+\rho \;s.t.\;h_{i}\neq h_{k}$$

(17) $$L_{ij}^{-}>\min_{}L_{ik}-\rho \;s.t.\;h_{i}=h_{k}$$

where ρ is a given margin. For anchor $s_{i}$, we denote the sets of sampled positive and negative pairs as $S_{i}^{+}$ and $S_{i}^{-}$, respectively.

#### 3.4.2. Image-Text Pairs Weighting

For the input image-text similarity matrix *L*, the gradients of the proposed AWL τ(L,ϑ) are as follows.

(18) $$\frac{\partial \tau (L,\vartheta )}{\partial \vartheta }=\sum_{i=1}^{B}\sum_{j=1}^{B}\frac{\partial \tau (L,\vartheta )}{\partial L_{ij}}\frac{\partial L_{ij}}{\partial \vartheta }$$

where ϑ are the model parameters to be learned, and *B* is the batch size in the training process. Notably, we define $W=\frac{\partial \tau (L,\vartheta )}{\partial L_{ij}}$ as a weight that indicates the role of each similarity in parameter optimization. In the following part, we elaborate on how to obtain different weights for different similarities.

In order to fully exploit the imbalance information existing in different pairs, we design two weighting schemes for the sampled positive and negative pairs, respectively. The weight between the anchor $s_{i}$ and the positive candidate $s_{j}$ is computed as:

(19) $$W_{ij}^{+}=\frac{e^{\left(1-L_{ij}\right)}}{\sum_{s_{k}}e^{\left(1-L_{ik}\right)}}\;s.t.\;s_{k}\in S_{i}^{+}$$

Similarly, the weight between the anchor $s_{i}$ and the negative candidate $s_{j}$ is calculated as:

(20) $$W_{ij}^{-}=\frac{e^{\left(L_{ij}-1\right)}}{\sum_{s_{k}}e^{\left(L_{ik}-1\right)}}\;s.t.\;s_{k}\in S_{i}^{-}$$

To integrate $W_{ij}^{+}$ and $W_{ij}^{-}$ into a unified representation, we introduce the indicator function 𝟙(·) in AWL.

(21) $$𝟙\left(x\right)=\left\{\begin{array}{cc} 1, & \mathrm{if}\;x\;\mathrm{is}\;\mathrm{true}\;\\ -1, & \mathrm{otherwise} \end{array}\right.$$

Afterwards, the weight between the anchor $s_{i}$ and the candidate $s_{j}$ is redefined as follows:

(22) $$W_{ij}=\frac{e^{𝟙\left(s_{k}\in S_{i}^{+}\right)\cdot \left(1-L_{ij}\right)}}{\sum_{s_{k}}e^{𝟙\left(s_{k}\in S_{i}^{+}\right)\cdot \left(1-L_{ik}\right)}}$$

where $s_{k}$ and $s_{j}$ both belong to positive candidates or negative candidates.

To improve the performance of image-text retrieval, larger weights are given to positive pairs with lower similarity, while larger weights are allocated to negative pairs with higher similarity. It is obvious that this strategy takes advantage of potential interactions among different pairs to learn the adaptive weights, which are used to optimize the image-text similarity. Finally, according to Equations (18)–(20), our proposed AWL is presented as follows.

(23) $$\tau \left(L,\vartheta \right)=\frac{1}{B}\sum_{i=1}^{B}\left\{\ln\left[\sum_{s_{k}\in S_{i}^{+}}e^{𝟙\left(s_{k}\in S_{i}^{+}\right)\cdot \left(1-L_{ij}\right)}\right]+\ln\left[\sum_{s_{k}\in S_{i}^{-}}e^{𝟙\left(s_{k}\in S_{i}^{+}\right)\cdot \left(1-L_{ij}\right)}\right]\right\}$$

Particularly, the gradient of τ(L,ϑ) with respect to $L_{ij}$ is calculated by judging as follows.

(24) $$\frac{\partial \tau (L,\vartheta )}{\partial L_{ij}}=\left\{\begin{array}{cc} -\frac{e^{\left(1-L_{ij}\right)}}{\sum_{s_{k}\in S_{i}^{+}}e^{\left(1-L_{ik}\right)}} & \mathrm{if}\;s_{j}\in S_{i}^{+}\\ \frac{e^{\left(1-L_{ij}\right)}}{\sum_{s_{k}\in S_{i}^{-}}e^{\left(1-L_{ik}\right)}} & \mathrm{if}\;s_{j}\in S_{i}^{-} \end{array}\right.$$

Especially, AWL is adopted in both the GAN module and the LAN module of HAAN.

## 4. Experiment

Experiments on 3 widely used cross-modality datasets will be conducted in this section. We compare their performance with 11 state-of-the-art methods, highlighting the advancement of HAAN. Furthermore, parameter sensitivity, convergence analysis and ablation studies are presented to demonstrate the effectiveness of HAAN and the contribution of each component in it.

### 4.1. Datasets

We briefly introduce three mainstream multi-modal datasets employed in our experiments, covering Corel 5K [16], Pascal Sentence [17] and Wiki [18], shown as below.

- Corel 5K dataset [16] totally contains 5000 images. It is composed of 50 semantic concepts, each of which involves 100 images. There are 260 tags attached to images in the dictionary, with an average of 1–5 tags for each image. Besides, images that are lacking tags are deleted. Finally, there are 4992 image-text pairs, in which the testing set and validation set consist of 249 pairs and 250 pairs, respectively, while the rest are regarded as the training set.
- Pascal Sentence dataset [17] consists of 1000 images. Each image is used to construct five sentences from different annotators to generate a single text. With 20 categories, the Pascal Sentence dataset is divided into three parts: 800 pairs for training, 100 pairs for validation, and 100 pairs for testing.
- Wiki dataset [18] collects 2866 images with corresponding texts for each, which describe characters, places or events. The content of the text is closely related to image information, constituting 2866 image-text pairs. Every pair can be classified into a certain category among 10 semantic concepts. Note that there are 2173 pairs in testing for training and 231 pairs in the validation set, while the rest are testing set.

### 4.2. Implementation Details

In this section, we provide details of model settings and training settings of HANN in this experiment.

#### 4.2.1. Simulation Parameters

Here are several experiments involving simulation parameters and descriptions in Table 2 to further assist in understanding the HAAN model.

#### 4.2.2. Model Settings

As we have mentioned in Section 3.2, α is set as 9. Specifically, we separate images into 3×3 patches in order to balance the computational cost and data capacity in local-level features extracting. As mentioned in Section 3.3, we refer to [7,19] and thus set λ as 9, and the sensitivity of parameters about HAAN is elaborated detailedly in Section 4.5.

#### 4.2.3. Training Settings

Our hierarchical alignment networks (i.e., GAN and LAN) are trained *E* epochs in a mini-batch by the Adam optimizer [20] with the batch size as *B*. It is worth noting that we normalize the common embedding features for each mini-batch by the $\ell_{2}$-norm as described in [21], which regularizes the model to prevent overfitting. More importantly, the maximum gradient norm is set to 2 to avoid gradient explosion for gradient clipping.

For all models on all datasets, we set the learning rate for the first E/2 epochs at 0.0002, and decrease it by 0.1 for the rest epochs. Particularly, the mini-batch size is set as 100 for Corel 5K with 100 epochs being considered; the bath size of Pascal Sentence is set as 10 with 30 epochs being utilized; the batch size for Wiki appears as 20 with 20 epochs. As these datasets contain training sets with different sizes, the quantity of iterations in each epoch is not fixed. We select the snapshot with the best result on the validation set for testing. At each epoch, our research assesses the efficacy of each model on the validation set to get the best model based on the mAP score. Next, we assess the best model for experimental results on the testing set. The HAAN approach is implemented by Pytorch [22] using the NVIDIA GeForce RTX 2080 GPU.

### 4.3. Evaluation Metric and Compared Methods

We perform image-text retrieval tasks on the above three datasets, and the tasks are divided into the following two types:

(1) Search text by image (I2T)
(2) Search image by text (T2I)

The mean Average Precision (mAP) is useful when testing the general performance of certain algorithms. The first step taken to work out mAP is to get the average precision (AP) of a set of *R* retrieved documents by Equation (25)

(25) $$AP=\frac{1}{T}\sum_{r=1}^{R}P\left(r\right)\times \delta \left(r\right)$$

here *T* represents how many relevant documents appear in the retrieved set, while P(r) means the precision of the top *r* retrieved documents. If the *r*th retrieved document turns out to be relevant (where relevant means belonging to the class of the query) then δ(r)=1, or δ(r)=0. Then, we average the AP values over all queries in the query set to calculate the mAP. Alternatively, methods with larger mAP turn out to be more effective. Apart from this, the precision-recall curve is another metric to measure the effectiveness of different methods. The PR curves show the varying trend of retrieval accuracy under all recall values. Similar to features of mAP, the curve that can enclose the larger area means a better result the model can achieve.

To confirm the effectiveness of HAAN, this research will make a comparison between HAAN and other 11 state-of-the-art methods, including 3 traditional methods, namely JRL [23], KCCA [24], JFSSL [25], and 8 deep learning methods, namely DCCA [26], SCAN [7], MAVA [27], SGRAF [28], SCL [29], CGMN [30], NAAF [10] and VSRN++ [31].

### 4.4. Comparison Results

Our HAAN method and 11 contrasting methods on all datasets are compared in terms of (1) I2T mAP scores, (2) T2I mAP scores and (3) mPA(AVG) scores (i.e., the average scores between (1) and (2)), as shown in Table 3. We use “∘” to mark the traditional method and exploit “•” to represent the deep learning method. In addition, the best results are shown in bold. From Table 3, we can easily find that HAAN achieves the best retrieval performance. Furthermore, HAAN improves the mAP(AVG) scores by 1.83 %, 1.20 % and 1.89 % respectively over the previous best model VSRN++ on Corel 5K, Pascal Sentence and Wiki. The performance of VSRN++ on I2T is better than that of HAAN, but only 0.57% higher, while HAAN can achieve similar high performance on both I2T and T2I, which indicates that HAAN is easier to solve practical problems.

**Figure 5:**
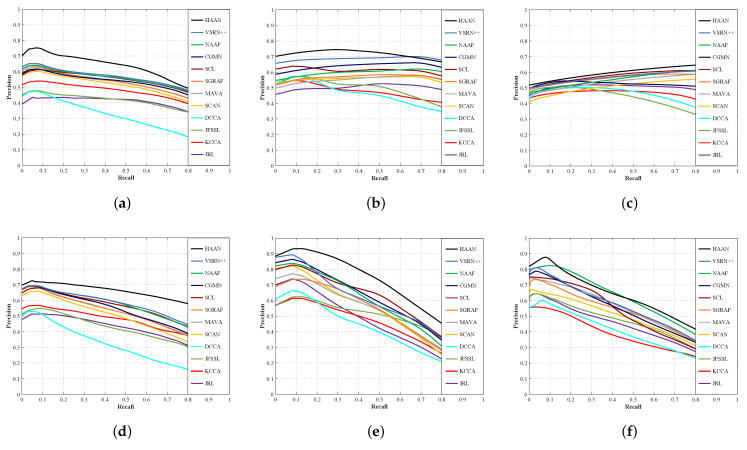
The PR curves of image-text retrieval for HAAN and other compared methods on all datasets. (**a**) I2T on Corel 5K; (**b**) I2T on Pascal Sentence; (**c**) I2T on Wiki; (**d**) T2I on Corel 5K; (**e**) T2I on Pascal Sentence; (**f**) T2I on Wiki.

It is worth noting that the text in a Pascal Sentence appears as a set of sentences, but in Corel 5K and Wiki, it is represented as a set of tags. Looking at mAP scores, HAAN performs better in image-text retrieval regardless of whether sentences or labels are used. We also find that the deep learning-based image-text retrieval methods perform better than traditional image-text retrieval methods. Next, the tasks of I2T and T2I are conducted on all datasets, and the PR curves are shown in Figure 5. From Figure 5, we can see that HAAN has the best overall performance because the area of the PR curve of HAAN tends to be larger than the area covered by the PR curves of other methods. Noticeably, VSRN++ is superior to HAAN only in the task of I2T in Pascal Sentence as shown in Figure 5c. However, HAAN is superior to VSRN++ in all other respects.

To better evaluate our method, we focus on the training time of deep learning methods to conduct a comparative experiment. Specifically, source codes of all the methods are implemented on the same machine with a single GPU. From Table 4, our findings go as follows. In the first place, DCCA and SCAN require the shortest training time, but perform less competitively than other deep learning methods in terms of image-text retrieval. Second, although MAVA, SGRAF, SCL, CGMN, NAAF have nearly the same training time as HAAN, HAAN outperforms them on image-text retrieval tasks. Finally, VSRN++ is secondary only to HAAN in image-text retrieval though it costs the longest training time.

Through a comprehensive analysis of these experimental results, conclusions can be summarized as follows:

(1) JRL, KCCA and JFSSL, the traditional image-text retrieval methods, are not as good as the image-text retrieval methods based on deep learning. Because deep neural networks can discover nonlinear image-text correlations.
(2) The attention mechanism-based model is significantly better than DCCA because it can effectively estimate image-text similarity by enabling latent matching between image patches and words. Specifically, SCAN computes the image-text similarity using visual regions and words as corresponding contexts. However, SCAN only exploits local-level relations. Different from SCAN, MAVA measures image-text similarity from the global, local, and relation levels, making it achieve better performance. Besides, SGRAF outperforms MAVA in suppressing uncorrelated interactions at the global and local levels. Furthermore, VSRN++ is secondary only to HAAN, but the training time of our HAAN is reduced by 16.04%, 16.01% and 14.29% compared with that of VSRN++ on the three data sets, respectively, which is very significant.
(3) SCL, CGMN and NAAF with outstanding performance can be observed, but they are not as good as HAAN. The reason is that these three methods do not consider the global-level information and the local-level information. Therefore, HAAN, which considers both global-level information and local-level information and further optimizes the two kinds of information, easily beats these three methods for roughly the same amount of training time.
(4) The comprehensive performance of HAAN is the best on all datasets. The reason is that HAAN can mine and fuse complementarities in multi-level data to cross the heterogeneous gap. Specifically, HAAN can accurately describe complex nonlinear image-text relationships, which is a distinct advantage over traditional methods. Since HAAN utilizes global-level and local-level information, it also significantly outperforms SCAN. Although both MAVA and SGRAF entirely use global-level and local-level data, HAAN keeps its advantages owing to the proposed AWL loss, which can accurately optimize image-text similarity by integrating pair mining and pair weighting in a unified framework.

In conclusion, HAAN fuses global-level and local-level information, and uses the proposed AWL to mine and enhance the two kinds of information, so the retrieval accuracy reaches the optimum. In addition, the first stage of AWL (i.e., image-text pairs sampling) selects valuable information while filtering redundant information, which accelerates the convergence and reduces the training time. HAAN achieves the effect of fast speed and high precision.

### 4.5. Parameter Sensitivity and Convergence Analyses

In this section, we conduct sensitivity analysis for the parameters, and convergence analysis for the hierarchical alignment network. The parameters involved in the proposed method is $\upsilon_{1}$ and $\upsilon_{2}$ mentioned in Section 3.1, ρ mentioned in Section 3.4.1. Besides, parametric sensitivity analyses are evaluated using mAP (AVG).

**Figure 6:**
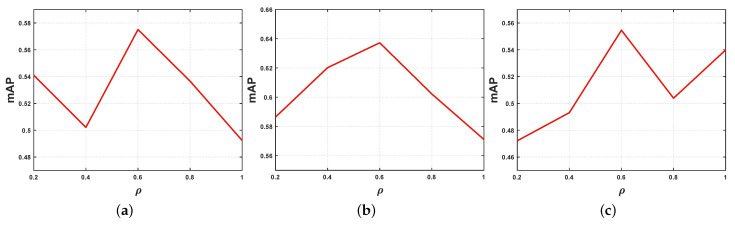
The mAP scores of HAAN in all datasets with ρ varying. (**a**) Corel 5K; (**b**) Pascal Sentence; (**c**) Wiki.

First, we set ρ to {0.2, 0.4, 0.6, 0.8, 1} and the experimental results are shown in Figure 6. It can be concluded that when ρ is 0.6, on the three selected datasets, the average mAP scores of I2T and T2I are the highest. To be specific, the highest scores of mAP on Corel 5K, Pascal Sentence and Wiki are 0.5751, 0.6410, and 0.5546, respectively.

**Figure 7:**
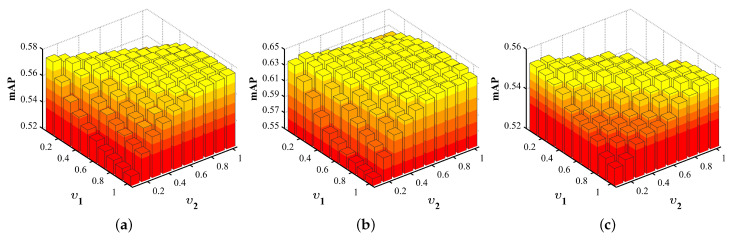
The mAP scores of HAAN in all datasets with $\vartheta_{1},\vartheta_{2}$ varying and ρ is fixed to the best value. (**a**) Corel 5K; (**b**) Pascal Sentence; (**c**) Wiki.

Second, we set $\upsilon_{1}$ and $\upsilon_{2}$ to {0.1, 0.2, 0.3, 0.4, 0.5, 0.6, 0.7, 0.8, 0.9, 1} and the experimental results are shown in Figure 7. According to the experimental results, when the ratio of $\upsilon_{1}$ and $\upsilon_{2}$ is 1:1, the mAP scores on the three datasets all reach the highest or are close to the highest, which proves that the importance of our two networks is basically the same. When the value of $\upsilon_{1}$ is fixed, the mAP value will first increase and then decrease as the value of $\upsilon_{2}$ increases from small to large. When the values of $\upsilon_{1}$ and $\upsilon_{2}$ are close to each other, the larger the mAP value will be, which confirms our conclusion above. When the value of $\upsilon_{2}$ differs greatly from that of $\upsilon_{1}$, the value of mAP decreases rapidly. This indicates that the complementarity of global-level information and local-level information is very necessary to enhance the performance of image-text retrieval.

**Figure 8:**
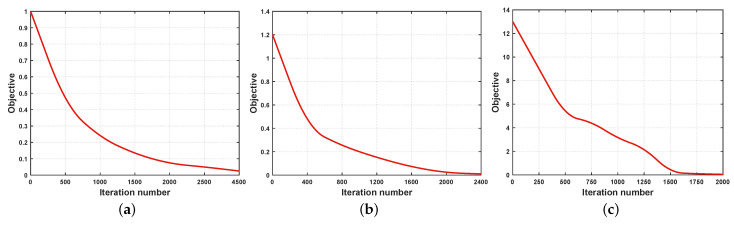
Convergence curves of ${ℶ}^{C}$ on all datasets. (**a**) Corel 5K; (**b**) Pascal Sentence; (**c**) Wiki.

Finally, the results of the convergence experiment for GAN are shown in Figure 8. We can easily observe that the objective function value of ${ℶ}^{C}$ monotonically decreases at each iteration. The reason lies in that our proposed AWL loss is effective. The convergence of LAN is not reported, because it is just similar to GAN.

### 4.6. Ablation Study

In this section, a series of ablation studies are conducted under different configurations of critical components of HAAN, in order to study the contribution of each component in the model.

As shown in Table 5, several models are provided for ablation studies to reveal the effectiveness of GCM, LCM, AWL (Stage 1) and AWL (Stage 2). Particularly, “∘” represents that the module (or loss function) is not contained in the model, while “•” denotes that the module (or loss function) is contained in the model. To further demonstrate the effectiveness of AWL, we combine it with Triplet loss (TRI) [12] for comparison. In the ablation model, TRI is used to replace AWL. We provide 7 combinations of the above 5 components, (e.g., HAAN-GCM represents HAAN with only the GCM module). The experimental results of our proposed ablation studies are shown in Table 6, from which the following conclusions can be drawn. Note that the best results in Table 6 are shown in bold.

- The map value of HAAN-LCM is 1.45%, 2.09%, and 1.7% higher than that of HAAN-GCM on Corel5K, Pascal sentence and Wiki, respectively. This is because the LAN captures more details through the attention mechanism to get more valuable information. The performance of HAAN-GCM-LCM is better than that of HAAN-GCM and HAAN-LCM. This shows that the local-level information is complementary and that better performance can be achieved by integrating the two networks (i.e., GCM and LCM).
- The mAP scores of HAAN-GCM-LCM-AWL (Stage 1) and HAAN-GCM-LCM-AWL (Stage 2) are very close, indicating that the two stages of AWL play almost the same importance in image-text similarity optimization. Furthermore, HAAN-GCM-LCM-AWL (Stage 1) and HAAN-GCM-LCM-AWL (Stage 2) are significantly better than HAAN-GCM-LCM-TRI. It is worth noting that the map value performed by any stage of the AWL shows higher than that of Triplet loss on three datasets by at least 1.3%, 2.63%, and 2.4%, respectively. This is due to the two stages of AWL addressing two major flaws of triplet loss respectively.
- The mAP score of HAAN is much higher than that of HAAN-GCM-LCM-AWL (Stage 1) and HAAN-GCM-LCM-AWL (Stage 2). This is because the integration of the two stages (i.e., AWL (Stage 1) and AWL (Stage 2)) compensates for the defect for a single stage of AWL. Specifically, (1) only HAAN-GCM-LCM-AWL (Stage 1) is used, and valuable samples are selected, but training samples with differences could not be fully used; (2) only HAAN-GCM-LCM-AWL (Stage 2) is used to optimize all samples with different strengths, but no redundant information is filtered out.
- From the column “AVG of all datasets”, we find that there are four main factors affecting system performance in our proposed HAAN, including (1) GCM, (2) LCM, (3) AWL (Stage 1), and (4) AWL (Stage 2). Specifically, the ways in which each factor affects the performance of image-text retrieval are shown below.
  (1) GCM: From a holistic perspective, it explores global-level alignment between the whole image and text to learn image-text similarity.
  (2) LCM: From the perspective of detail, explore the local alignment of image patches and key words, and learn the similarity of images and texts.
  (3) AWL (Stage 1): Select valuable sample pairs (i.e., (1) Select samples far away from the anchor point to generate positive pairs; (2) select samples close to the anchor point to generate negative pairs ) and filter redundant pairs.
  (4) AWL (Stage 2): Make full use of discriminant training samples and assign suitable weight to each positive and negative pair to achieve adaptive optimization.

To further verify the effectiveness of each factor, we conduct a series of ablation studies in the experiment. Furthermore, we add a new column titled “AVG of all datasets” in Table 6. First of all, it can be convinced that the performances of HAAN-GCM and HAAN-LCM are close to each other, which proves that fine-grained data and coarse-grained data are of equal importance in the task of image-text retrieval. The values of HAAN-GCM-LCM-AWL (Stage 1) and HAAN-GCM-LCMAWL (Stage 2) are approximately the same, meaning that the effect of Stage 1 and Stage 2 are almost equal. Furthermore, we can observe that when a certain stage of AWL is added, the performance of HAAN is improved by about 3% compared with that of the global-level or local-level alone. The huge improvement it involves shows that AWL is very effective in promoting the performance of HAAN. At the same time, it is clearly shown that the performance of HAAN is about 2% better than using AWL in a certain stage alone, making sure the efficiency of aggregation of the two modules.

From an overall point of view, all these four modules attach great importance. GCM and LCM lay the foundation for subsequent optimization and further improvement of the model. AWL, when dealing with fully aggregated information (i.e., global-level, and local-level information), can quickly improve the overall performance of the model. When two stages are employed together, the optimization effect of AWL improves by more than about 4% compared to TRI. This also confirms the remarkable optimization effect of AWL, performing a much better result. Further, for your convenience, we have listed the main contents below.

In conclusion, we can draw the following conclusions: (1) when performing the task of image-text retrieval, each component in HAAN plays a positive role; (2) HAAN effectively mines and fuses complementarity in multi-granularity data, which can provide essential clues for bridging the heterogeneous gap.

### 4.7. Qualitative Results

**Figure 9:**
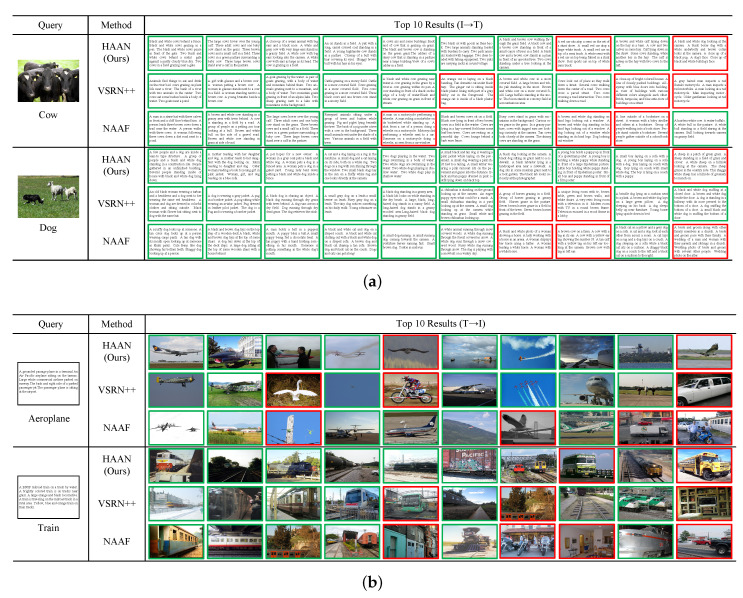
Example results for I2T and T2I on Pascal Sentence dataset using HAAN, VSRN++ and NAAF. Specifically, the green border indicates a correct search result, and the red border indicates an incorrect search result. (**a**) Examples of I2T retrieval results on Pascal Sentence dataset; (**b**) Examples of T2I retrieval results on Pascal Sentence dataset.

We provide typical examples of image-text retrieval on the Pascal Sentence dataset by two state-of-the-art image-text retrieval methods (i.e., VSRN++ and HAAN) as well as HAAN. It shows the top ten results for I2T and T2I correspondences for a specific query. In particular, in Figure 9a, we select two queries of the I2T for retrieval of “cow” and “dog”. In Figure 9b, we select two queries on T2I for retrieval of “aeroplane” and “train”.

For the task I2T, HAAN shows the best performance because its query results have the fewest errors. It is worth noting that, as in the retrieval of “cow”, the error text still contains some correct words (e.g., “black”, “white” and “face”) that match the correct semantic information in the query image.

Furthermore, for the task T2I, VSRN++ and NAAF all make more mistakes. At the same time, HAAN obtains the results with the fewest mistakes, which partially deviate from the semantic information but contain features similar to correct semantic information. For example, images of birds in flight appear in the retrieval of “aeroplane”. In contrast, VSRN++ and NAAF get more errors and deviate from the correct semantic information largely.

From this, we can conclude that HAAN significantly outperforms VSRN++ and NAAF when performing tasks I2T and T2I. It should be noted that NAAF is the worst performer among the three methods, not only for the much more wrong results it returns in both retrieval tasks but also for the semantic concept of wrong results that are totally different from the correct semantic information. For example, when searching for “aeroplane”, the search results show pictures of motorcycles and trucks; when searching for “train”, the search results show pictures of buildings and interiors of rooms. These search results that seriously deviate from the correct semantic information are entirely unacceptable. It shows that the performance of the NAAF is the worst one.

All in all, HAAN is superior to the two most advanced methods, achieving the best performance.

## 5. Conclusions

In this paper, we put forward HAAN to explore image-text alignment. First, hierarchical alignment networks (i.e., GCM and LCM) are proposed to exploit the rich complementary information in global-level and local-level features for image-text correlation learning. Secondly, our AWL integrates pairs mining and pairs weighting to optimize image-text similarity calculated from two modules (i.e., GCM and LCM). Experimental results show that our proposed HAAN achieves the optimal achievement in image-text retrieval tasks, and each component of HAAN is proven to be effective.

In the future, we will try more levels of alignment, and verify the scalability of HAAN (i.e., cross-modality for other types of modalities for retrieval tasks, (e.g., video queries text)) for more practical applications.

## Figures and Tables

**Table 1:** Important notations and descriptions.

Notation	Description
τ	the adaptive weighted loss
${ℶ}^{C}$	the global-level objective function
${ℶ}^{F}$	the local-level objective function
$\vartheta^{C}$	the parameters of GAN
$\vartheta^{F}$	the parameters of LAN
$L^{C}$	the global-level similarity matrix
$L^{F}$	the local-level similarity matrix
${\tilde{L}}^{C}$	the optimal global-level similar matrix
${\tilde{L}}^{F}$	the optimal local-level similar matrix
$\tilde{L}$	the matrix used to perform image-text retrieval
$\upsilon_{1}$	the global-level fusion coefficient
$\upsilon_{2}$	the local-level fusion coefficient
$n_{p}^{C}$	the global-level feature vector of image
$m_{q}^{C}$	the global-level feature vector of text
$N_{p}^{F}$	the local-level feature vector of image
$M_{q}^{F}$	the local-level feature vector of text
$L_{ij}^{+}$	the similarity between the samples of a positive pair
$L_{ij}^{-}$	the similarity between the samples of a negative pair
$W_{ij}^{+}$	the weight of sampled positive pair
$W_{ij}^{-}$	the weight of sampled negative pair

**Table 2:** Simulation parameters and descriptions.

Parameters	Description
α	the number of image patches segmented by each image
λ	the temperature-inverse parameter for the softmax function
*E*	the training epochs of our hierarchical alignment networks
*B*	the batch-size of Adam optimizer
$\vartheta^{C}$	the parameters of GAN
$\vartheta^{F}$	the parameters of LAN
ρ	the margin to assist in the sampling of pairs

**Table 3:** The mAP scores of image-text retrieval for HAAN and other compared methods on all datasets.

Methods	Corel 5K			Pascal Sentence			Wiki		
	*I*2*T*	*T*2*I*	AVG	*I*2*T*	*T*2*I*	AVG	*I*2*T*	*T*2*I*	AVG
∘ JRL (2013)	0.4081	0.4197	0.4139	0.5208	0.5067	0.5138	0.3390	0.2500	0.2945
∘ KCCA (2014)	0.4419	0.4503	0.4461	0.4889	0.4463	0.4676	0.4363	0.3891	0.4127
∘ JFSSL (2015)	0.4141	0.4139	0.4140	0.5073	0.4640	0.4856	0.3607	0.2801	0.3204
• DCCA (2013)	0.3107	0.3064	0.3086	0.4754	0.4719	0.4737	0.4400	0.3960	0.4150
• SCAN (2018)	0.4916	0.4886	0.4901	0.5662	0.5709	0.5686	0.5173	0.4395	0.4784
• MAVA (2019)	0.5217	0.5134	0.5176	0.5723	0.5711	0.5717	0.5475	0.4888	0.5181
• SGRAF (2021)	0.5241	0.5136	0.5189	0.5876	0.5727	0.5802	0.5644	0.4830	0.5237
• SCL (2022)	0.5404	0.5501	0.5453	0.6185	0.6219	0.6202	0.5637	0.4901	0.5269
• CGMN (2022)	0.5266	0.5231	0.5249	0.6218	0.6059	0.6139	0.5697	0.4911	0.5304
• NAAF (2022)	0.5493	0.5538	0.5516	0.6156	0.6286	0.6211	0.5699	0.4981	0.5340
• VSRN++ (2022)	0.5589	0.5546	0.5568	**0.6475**	0.6104	0.6290	0.5744	0.4970	0.5357
• HAAN (ours)	**0.5703**	**0.5799**	**0.5751**	0.6418	**0.6403**	**0.6410**	**0.5860**	**0.5232**	**0.5546**

**Table 4:** Training time of our HAAN method and comparison methods on all datasets.

Methods	Corel 5K	Pascal Sentence	Wiki
DCCA	8732 s	2177 s	3677 s
SCAN	12,656 s	3460 s	4997 s
MAVA	20,032 s	4351 s	5789 s
SGRAF	27,959 s	5024 s	6420 s
SCL	25,334 s	3851 s	4761 s
CGMN	32,217 s	6019 s	7640 s
NAAF	26,839 s	4596 s	6031 s
VSRN++	34,973 s	6579 s	8279 s
HAAN	29,363 s	5526 s	7096 s

**Table 5:** Experimental configurations of different ablative models.

Model	Module		Loss Function		
	GCM	LCM	AWL		TRI
			Stage 1	Stage 2	
HAAN-GCM	•	∘	∘	∘	∘
HAAN-LCM	∘	•	∘	∘	∘
HAAN-GCM-LCM	•	•	∘	∘	∘
HAAN-GCM-LCM-TRI	•	•	∘	∘	•
HAAN-GCM-LCM-AWL (Stage 1)	•	•	•	∘	∘
HAAN-GCM-LCM-AWL (Stage 2)	•	•	∘	•	∘
HAAN	•	•	•	•	∘

**Table 6:** Experimental results of ablation study on all datasets with mAP scores.

Model	Corel 5K			Pascal Sentence			Wiki			AVG of All Datesets
	*I*2*T*	*T*2*I*	AVG	*I*2*T*	*T*2*I*	AVG	*I*2*T*	*T*2*I*	AVG	
HAAN-GCM	0.4985	0.5083	0.5034	0.5347	0.5311	0.5329	0.5024	0.4310	0.4667	0.5010
HAAN-LCM	0.5164	0.5194	0.5179	0.5564	0.5512	0.5538	0.5196	0.4478	0.4837	0.5185
HAAN-GCM-LCM	0.5298	0.5310	0.5304	0.5739	0.5689	0.5714	0.5310	0.4602	0.4956	0.5325
HAAN-GCM-LCM-TRI	0.5371	0.5401	0.5386	0.5932	0.5904	0.5918	0.5472	0.4712	0.5092	0.5465
HAAN-GCM-LCM-AWL (Stage 1)	0.5523	0.5511	0.5517	0.6146	0.6216	0.6181	0.5693	0.4971	0.5332	0.5677
HAAN-GCM-LCM-AWL (Stage 2)	0.5542	0.5576	0.5559	0.6195	0.6211	0.6203	0.5671	0.5043	0.5357	0.5706
HAAN	**0.5703**	**0.5799**	**0.5751**	**0.6418**	**0.6403**	**0.6410**	**0.5860**	**0.5232**	**0.5546**	0.5902

## Data Availability

Data will be made available on request.

## Abbreviations

The following abbreviations are used in this manuscript:

HAAN	Hierarchical Adaptive Alignment Network
CNN	Convolutional Neural Networks
GCM	Global-level Image-Text Similarity Computation Module
LCM	Local-level Image-Text Similarity Computation Module
AWL	Adaptive Weighted Loss
AWL (Stage 1)	the first stage of AWL
AWL (Stage 2)	the second stage of AWL
GAN	Global Alignment Network
LAN	Local Alignment Network
Bi-GRU	Bidirectional Gated Recurrent Units
AVG	The average mAP scores of I2T and T2I
TRI	Triplet loss
HAAN-GCM	HANN only use GCM
HAAN-LCM	HAAN only use LCM
HAAN-GCM-LCM	HAAN only use GCM and LCM
HAAN-GCM-LCM-TRI	HAAN only use GCM, LCM and TRI
HAAN-GCM-LCM-AWL (Stage 1)	HAAN only use GCM, LCM and AWL (Stage 1)
HAAN-GCM-LCM-AWL (Stage 2)	HAAN only use GCM, LCM and AWL (Stage 2)
